# Medial Meniscus Posterior Root Tears Associated With Medial Collateral Ligament Injury in Young and Middle‐Aged Adults: A Report of Three Cases

**DOI:** 10.1155/cro/9938580

**Published:** 2026-06-29

**Authors:** Goki Kamei, Ayaka Yamazato, Atsushi Takazawa, Hiroo Hiramatsu

**Affiliations:** ^1^ Department of Orthopaedic Surgery, Hiroshima Hiramatsu Hospital, Hiroshima, Japan

**Keywords:** arthroscopic repair, knee trauma, medial collateral ligament injury, medial meniscus posterior root tear, young adults

## Abstract

Medial meniscus posterior root tears (MMPRTs) are increasingly recognized as a significant cause of rapid medial compartment degeneration. Although commonly observed in middle‐aged patients with degenerative changes, traumatic MMPRTs in younger individuals are uncommon and may be overlooked, particularly when occurring alongside medial collateral ligament (MCL) injuries. Herein, we report three cases of MMPRTs associated with Grades I–II MCL injuries in young to middle‐aged adults. All patients sustained a valgus force during athletic activity and were initially diagnosed with isolated MCL injuries based on early magnetic resonance imaging (MRI). Upon referral to our institution, persistent medial joint pain prompted a repeat MRI, which revealed concomitant MMPRTs. Surgical repair was performed 3–4 months postinjury using circumferential fiber augmentation with either transtibial pullout or centralization techniques. Postoperative MRI at 6–12 months showed no progression of meniscal extrusion. Two patients returned to sports, and one discontinued follow‐up due to pregnancy. These cases emphasize the importance of considering MMPRT in young patients presenting with MCL injuries. Early treatment is most important to avoid delayed diagnosis and prevent cartilage degeneration.

## 1. Introduction

Medial meniscus posterior root tears (MMPRTs) disrupt the circumferential hoop tension essential for load distribution in the medial compartment. The loss of this mechanism results in biomechanical conditions equivalent to total meniscectomy, leading to increased contact pressures and accelerated cartilage degeneration. Allaire et al. [1] reported a 25% increase in the medial compartment contact pressure following MMPRT, comparable with complete meniscal removal. Although degenerative MMPRTs are common in middle‐aged and older individuals, traumatic MMPRTs in young adults are relatively rare and reported with multiligament injury [2]. Kim et al. [2] reported a 2.74% incidence of MMPRTs in multiligament knee injuries, with no cases involving isolated MCL injuries. Medial collateral ligament (MCL) injuries are among the most frequent sports‐related knee injuries. Grades I and II injuries generally respond well to conservative treatment, with a rapid return to activity [3]. On the contrary, MMPRTs in young adult patients usually require surgical treatment because MMPRTs cause cartilage injury, osteoarthritis, and osteonecrosis. This report presents three cases of MMPRTs associated with Grades I–II MCL injuries in young to middle‐aged patients.

## 2. Case Presentation

Three patients—an adolescent male (aged 16 years), a young adult male (aged 26 years), and an adult female (aged 35 years)—presented with medial knee pain following sports‐related trauma. The data of the patients are shown in Table 1.

**Table 1:** Patient data.

	Age	Gender	Height (cm)	Weight (kg)	Sports	FTA in standing position	%MA in standing position	MTPA
Case 1	16	Male	170.0	57.5	Soccer	181.2°	47.4%	86.6°
Case 2	26	Male	174.7	81.2	Basketball	187.1°	11.5%	81.7°
Case 3	35	Female	155.0	44.0	Soccer	178.2°	49.5%	85.4°

Abbreviations: FTA, femorotibial Angle; MA, mechanical axis; MTPA, medial tibial plateau angle.

### 2.1. Case 1: 16‐Year‐Old Boy

**Figure 1:**
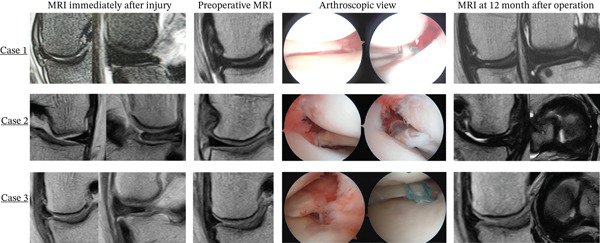
MRI and arthroscopic findings. Left: MRI immediately after injury showed MCL injury and MMPRTs. Middle: Preoperative MRI showed the progression of extrusion of the medial meniscus. Arthroscopic view showed the MMPRTs (left) and the image after the repair (right). Right: MRI at 12 months after operation showed the posterior root repair and the improvement of extrusion of the medial meniscus.

The injury occurred during dribbling motions in soccer when a direct valgus force was applied to the knee. The angle of knee flexion is estimated to be approximately 30°. The following day, the patient visited a local clinic and was diagnosed with an MCL injury; conservative treatment was recommended. He was fitted with a hinged brace and underwent treatment focused on strength training and range‐of‐motion (ROM) exercises for 1 month. One month after the injury, supervised physiotherapy training began to return to soccer, but pain on the medial side of his knee persisted and gradually worsened. Concerned about the ongoing pain, he then visited another hospital, where an initial magnetic resonance imaging (MRI) revealed a concomitant injury to the posterior root of the medial meniscus. Due to the complexity of the injury, he was referred to our hospital for surgical treatment. On physical examination at the initial visit, the ROM was not limited. There was severe tenderness of the medial joint space. There was no significant valgus instability. MRI at the time of injury showed an MCL injury at the femoral attachment and posterior root tear of the medial meniscus. An MRI performed at our institution demonstrated complete MMPRT (Figure 1). Mild medial meniscal extrusion was also observed, showing slight progression compared with imaging obtained immediately after the injury (Table 2).

**Table 2:** Extrusion of the medial meniscus on MRI.

	Immediately after injury (mm)	Preoperation (mm)	At 12 month after operation (mm)
Case 1	2.14	3.75	3.14
Case 2	2.50	5.01	4.38
Case 3	3.75	4.10	3.13

### 2.2. Case 2: 26‐Year‐Old Male

The patient sustained a knee injury during a basketball game due to a direct valgus force while dribbling. Initially, the knee was flexed at approximately 30°. On the day of the injury, he visited a local clinic where an MRI revealed an MCL injury at the femoral attachment and MMPRTs. The MCL injury was managed conservatively with rest and hinged bracing, but the MMPRTs were initially overlooked. After 1 month of strength training and ROM exercises, his pain worsened upon attempting supervised rehabilitation. Subsequent MRIs showed a posterior root tear of the medial meniscus and meniscal displacement, necessitating surgical intervention. Two months postinjury, he was referred to our hospital due to persistent medial pain and limited function. Initial evaluations showed no significant valgus instability, but tenderness was localized to the medial joint line. The McMurray tests indicated pain without mechanical locking, and the ROM was not limited. An MRI at our institution confirmed a complete radial MMPRT and mild medial meniscal extrusion, prompting surgery 3 months postinjury. Arthroscopy confirmed posterior root disruption (Type II), and he underwent circumferential fiber augmentation (CFA) combined with transtibial pullout repair.

### 2.3. Case 3: 36‐Year‐Old Female

She sustained a knee injury while playing soccer. The injury occurred during dribbling motions when a direct valgus force was applied to the knee. The angle of knee flexion is estimated to be approximately 30°. Initial MRI at previous clinics revealed an MCL injury at the femoral attachment and MMPRTs. She was diagnosed with an MCL injury managed conservatively with rest and hinged bracing. MMPRTs were overlooked. She was fitted with a hinged brace and underwent treatment focused on strength training and ROM exercises for 1 month. She experienced increased pain during daily activities. At 3 months after the injury, she was referred to our hospital for persistent medial pain in daily life. At presentation, no significant valgus instability was detected, and tenderness was localized to the medial joint line. The McMurray tests elicited pain without mechanical locking. The ROM was not limited. An MRI taken immediately after the injury demonstrated the femoral‐sided MCL injury and MMPRT (Figure 1). An MRI performed at our institution demonstrated complete radial MMPRT (Figure 1). Mild medial meniscal extrusion was also observed, showing only a very slight progression compared with imaging obtained immediately after the injury (Table 2).

### 2.4. Surgical Treatment

Arthroscopy confirmed posterior root disruption (Type II) in all cases. The manual valgus stress test under anesthesia revealed no valgus instability in either the extended or mildly flexed positions, and intraoperative arthroscopic confirmation showed no MCL instability, leading us to conclude that there was no instability due to an MCL injury. Therefore, we performed treatment on the posterior root of the medial meniscus only. Cases 1 and 2 underwent CFA combined with transtibial pullout repair at 3 months after injury, whereas Case 3 received CFA along with a meniscal centralization technique at 4 months after injury. CFA was performed according to the technique described by Kita et al. [4]. Centralization was performed using two Knotless 1.8‐mm FiberTak soft anchors to reduce the meniscus deviation of medial meniscus.

### 2.5. Postoperative Rehabilitation and Outcomes

The knee was immobilized at extension position for 2 weeks. Thereafter, active and passive motion exercises were encouraged. Knee flexion exercise was limited to 90° for the first 4 weeks. The patient was allowed 120° knee flexion after 4 weeks. Deep knee flexion was permitted 3 months postoperatively. The patients were allowed partial weight‐bearing at 2 weeks and full weight‐bearing at 5 weeks after surgery. Jogging was initiated at 3 months, agility training was started at 4.5 months, and return to sports was permitted at 6 months. The clinical outcomes varied among the cases as follows: Case 1 returned to competitive sports (Tegner activity score: 10), Case 2 resumed recreational sports (Tegner activity score: 8), and Case 3 discontinued sports activity due to pregnancy. Follow‐up MRI (12 months) showed that meniscal extrusion has shown little to no progression and satisfactory healing of the repair site in all cases (Figure 1). Patient‐reported outcome measures was evaluated by Knee Injury and Osteoarthritis Outcome Score (KOOS) scores. KOOS score improved dramatically 1 year after surgery in Cases 1 and 2. KOOS symptom score (60.7–96.4, 57.1–82.1), pain score (61.1–100, 69.4–94.4), ADL score (83.3–100, 82.4–100), QOL score (40–100, 40–95), and SP score (25–100, 31.3–93.8) (Cases 1 and 2).

## 3. Discussion

The key finding in this case series is that MMPRTs can complicate low‐grade MCL injuries even in young and middle‐aged patients. Although MCL injuries alone are typically managed conservatively, the presence of MMPRT significantly changes the prognosis and requires surgical intervention. Therefore, clinicians should ensure careful assessment of the meniscal root whenever an MCL injury is diagnosed in young individuals.

The frequency of MMPRT in multiple ligament injuries is low (2.74%), and its association with isolated MCL injury has not been well‐documented [2]. This is the first report of a combined injury involving an MCL tear and posterior root tear of the medial meniscus. The mechanism in all three cases involved a valgus force applied during weight‐bearing. This suggests that although the MCL absorbs valgus stress, compressive overload of the medial compartment during trauma may injure the meniscal root. Importantly, the MCL injury grade and morphology did not correlate with MMPRT occurrence. All tears were Grades I–II with no specific pattern, indicating that MMPRT may occur independently of MCL severity.

Diagnosis is difficult for the following reasons. First, the pain from MCL and MMPRT tend to overlap. Second, low‐grade MCL injuries regain stability quickly, and the pain with MMPRT often disappears quickly, masking deeper pathology. Finally, since there are no past reports of isolated MCL tears combined with posterior root meniscus tears in cases without ACL injury, there is a potential for misdiagnosis. The final reason is the most important, and we must keep in mind that MCL injury may be complicated by MM posterior root injury.

The treatment must address both injuries. For the MCL injury (Grades I–II), conservative management is standard, including bracing, activity modification, ROM exercises, and progressive strengthening [3, 5]. Biomechanically, MMPRTs abolish hoop function and increase medial compartment pressure by 25%, equivalent to total meniscectomy [1]. Untreated MMPRTs predispose patients to a rapid progression of cartilage damage, subchondral collapse, and osteoarthritis [6]. It has been reported that in patients aged 50 or older, results are poor when relying solely on MMPRTs repair, and alignment correction using methods such as OWHTO is necessary. If alignment correction is to be performed, it must be corrected to 60% or higher [7, 8]. In younger patients, surgical repair is associated with favorable outcomes, especially in those under 50 years of age [8]; therefore, arthroscopic root repair is recommended. In this case, because the patients were young—aged 16, 25, and 35—no lower limb alignment correction was performed, and good results were achieved. The techniques used in these cases (CFA, pullout, and centralization) have shown promising results in restoring the meniscal function and preventing extrusion [4, 9, 10]. In this case series, there was no progression of meniscal extrusion on MRI at 6–12 months after surgery. Notably, surgery should be performed as soon as possible after injury. These cases illustrate important considerations, showing that traumatic MMPRTs can occur even in young patients with seemingly mild MCL injuries, that persistent medial knee pain after an MCL injury should be regarded as a red flag, and that early surgical repair is crucial for preventing long‐term cartilage degeneration. Since isolated MCL injuries of Grade 1or II are common in sports‐related injuries, it is believed that the diagnosis of MCL injury alone in these three cases was reached based on the preconception that they were isolated MCL injuries. Clinicians must keep in mind the possibility that a mild MCL injury may be complicated by a posterior root injury of the medial meniscus.

## 4. Conclusion

It is important to keep in mind that isolated MCL injuries, which are candidates for conservative treatment in Grades 1 and 2, may be complicated by posterior root meniscus tears.

## Funding

No funding was received for this manuscript.

## Consent

Written informed consent was obtained from the patient prior to the induction of this study.

## Conflicts of Interest

The authors declare no conflicts of interest.

## Data Availability

The data that support the findings of this study are available on request from the corresponding author. The data are not publicly available due to privacy or ethical restrictions.
